# Dried shiitake mushroom grade recognition using D-VGG network and machine vision

**DOI:** 10.3389/fnut.2023.1247075

**Published:** 2023-10-18

**Authors:** Li Wang, Penghao Dong, Qiao Wang, Kunming Jia, Qunfeng Niu

**Affiliations:** ^1^School of Electrical Engineering, Henan University of Technology, Zhengzhou, China; ^2^Key Laboratory of Grain Information Processing and Control, Ministry of Education, Henan University of Technology, Zhengzhou, China

**Keywords:** dried shiitake mushroom, grading, image preprocessing, deep learning, classification model

## Abstract

Grading dried shiitake mushrooms is an indispensable production step, as there are large quality differences between different grades, which affect the product’s price and marketability. Dried shiitake mushroom samples have irregular shapes, small morphological differences between different grades of the same species, and they may occur in mixed grades, which causes challenges to the automatic grade recognition using machine vision. In this study, a comprehensive method to solve this problem is provided, including image acquisition, preprocessing, dataset creation, and grade recognition. The osprey optimization algorithm (OOA) is used to improve the computational efficiency of Otsu’s threshold binarization and obtain complete mushroom contours samples efficiently. Then, a method for dried shiitake mushroom grade recognition based on the improved VGG network (D-VGG) is proposed. The method uses the VGG16 network as the base framework, optimizes the convolutional layer of the network, and uses a global average pooling layer instead of a fully connected layer to reduce the risk of model overfitting. In addition, a residual module and batch normalization are introduced to enhance the learning effect of texture details, accelerate the convergence of the model, and improve the stability of the training process. An improved channel attention network is proposed to enhance the feature weights of different channels and improve the grading performance of the model. The experimental results show that the improved network model (D-VGG) can recognize different dried shiitake mushroom grades with high accuracy and recognition efficiency, achieving a final grading accuracy of 96.21%, with only 46.77 ms required to process a single image. The dried shiitake mushroom grade recognition method proposed in this study provides a new implementation approach for the dried shiitake mushroom quality grading process, as well as a reference for real-time grade recognition of other agricultural products.

## 1. Introduction

The shiitake mushroom is an excellent edible mushroom with a long history, and is one of the first domesticated and cultivated mushrooms. It is rich in nutrients and active substances (1, 2), and has medicinal and dietary uses, including cholesterol- and blood pressure-lowering effects; antibacterial, antifungal, antiviral, and antioxidant properties; it regulates intestinal microbes and enhances the immune system (3, 4). These properties are the reason for which it is known as the “king of mushrooms.” In recent years, the demand for shiitake mushrooms has been increasing significantly. Due to the influence of soil environment and light and temperature during growth, the surface of the shiitake mushroom cap usually cracks, forming a characteristic texture with different colors and patterns (5). Generally, this texture is whitish-brown and resembles the shape of a daisy, and such samples are known as “dried flower mushrooms.” In contrast, mushrooms without cracks on the surface of the cap are called “dried thick mushrooms” and “dried thin mushrooms.” Depending on their appearance and texture, different types of dried shiitake mushrooms (DSM) are classified into three grades: premium, first grade, and second grade, where a higher grade corresponds to better quality. The quality of shiitake mushrooms is the key factor to identify their grade during the acquisition process. Therefore, there is an urgent need to develop an efficient and accurate automatic grading method to ensure the accurate classification of the mushrooms according to their quality and achieve rapid grading.

In recent years, deep learning (6) has created a research boom in various fields. In the agricultural field, deep learning combined with machine vision has been widely used in plant recognition and detection, such as recognition of wood categories (7), fruit and vegetable classification (8–10), plant pest and disease identification (11, 12), crop yield estimation (13, 14) and weed detection (15).

Meanwhile, deep learning is being rapidly adopted in tasks related to agricultural product quality grading. Zhang et al. (16) proposed a peanut pod grade recognition method based on an improved AlexNet model with transfer learning. To train the model, the initial dataset of 500 collected images of five grades of peanut pods was enhanced to 3,500 images, and the final average recognition accuracy was 95.43%. Ren et al. (17) proposed a pepper quality detection method based on a convolutional neural network (CNN) and transfer learning. The method performed the grading based on the appearance features of peppers, and achieved a prediction accuracy of 98.14%. Lu et al. (18) proposed a grading method for roasted tobacco based on an improved ResNet50 network with multi-scale feature fusion. The method was tested on a total of 6,068 tobacco images from 7 grades and the final grading accuracy was 80.14%. Chen et al. (19) proposed an intelligent grading method for pecan kernels based on deep learning and physiological indicators, with a dataset of 4,395 images of pecan kernels of four grades enhanced to 6,213 images and a final grading accuracy of 92.2% on the test set.

In the research on DSM grading, initially, the identification of grades mainly relied on manual sorting, weighing, and mechanical classification methods based on sieve pore screens. Manual sorting involves the grade estimation by experienced workers; however, this method is less efficient, and the grading is subject to individuals’ subjective assessment, resulting in higher sorting error and lower grading accuracy. The weighing method involves sorting the DSM grade through its weight; although this method has improved in efficiency, it has limitations because it does not take into account the texture characteristics of the top cap to distinguish between grades. Mechanical grading based on sieve holes grades the DSM using different sieve hole sizes. This method is fast but also does not take into account the texture characteristics of DSM caps. In addition, the shapes of some DSM may not be consistent with the fixed size of sieve holes of the corresponding grade, which leads to grading inaccuracy.

With the development of machine vision and deep learning technologies in agricultural products, some domestic and foreign scholars have realized the identification of different grades through an analysis of DSM image features. Chen et al. (20) extracted suitable texture areas from the surface of DSM caps, used grayscale histogram statistics, a grayscale co-occurrence matrix, a Gaussian Markov random field model, and a fractal dimension model for feature extraction, and designed a *k*-nearest neighbor classifier to classify the image features with a correct classification rate of 93.57%. Shi et al. (21) designed a method for classifying DSM based on the texture features of cap openings, and established a quality factor calculation equation, with the final classification accuracy reaching 94.18%. Ketwongsa et al. (22) proposed a deep learning model for the classification of toxic and edible shiitake mushrooms based on an improved AlexNet CNN, and the accuracy of the proposed model reached 98.50% and 95.50%, respectively. What’s more, some intelligent mushroom sorting lines have been reported in field applications, such as the Wuhan Cooper smart mushroom sorting line.

Although some achievements have been made in mushroom species detection and quality grading, there are still some limitations. First, traditional machine vision methods are slow in the grading process and not adapted to the requirements of actual field environments. Second, most of the existing research focuses on the classification of different kinds of mushrooms, and there is less research on different grades of the same kind of mushrooms. This leads to many challenges in practical applications, such as fast implementations, algorithms optimization, model generalization and performance reliability.

In this study, a complete image processing method and grading scheme are proposed based on machine vision and deep learning for online real-time identification of different DSM grades. The method achieves accurate and efficient identification of different kinds and grades of DSM. The main contributions of this study are as follows:

1. An improved Otsu’s threshold binarization (OOA-Otsu) algorithm is designed to segment the DSM images by extracting the maximum contour and cropping the maximum contour external matrix. Then, the complete DSM image is obtained by extending the region of interest (ROI). An image dataset containing 1,355 original DSM of different grades was created.
2. A D-VGG network is constructed using the VGG16 network as the basic framework. The network can identify different grades of DSM effectively through its optimized convolutional layer and through the adoption of a global average pooling layer instead of a fully connected layer, the addition of a residual module and batch normalization, and fusion *via* an improved channel attention network. The accuracy rate achieved on the DSM dataset was 96.21%, which is better than other similar deep learning methods.

## 2. Image acquisition and preprocessing

**Figure 1:**
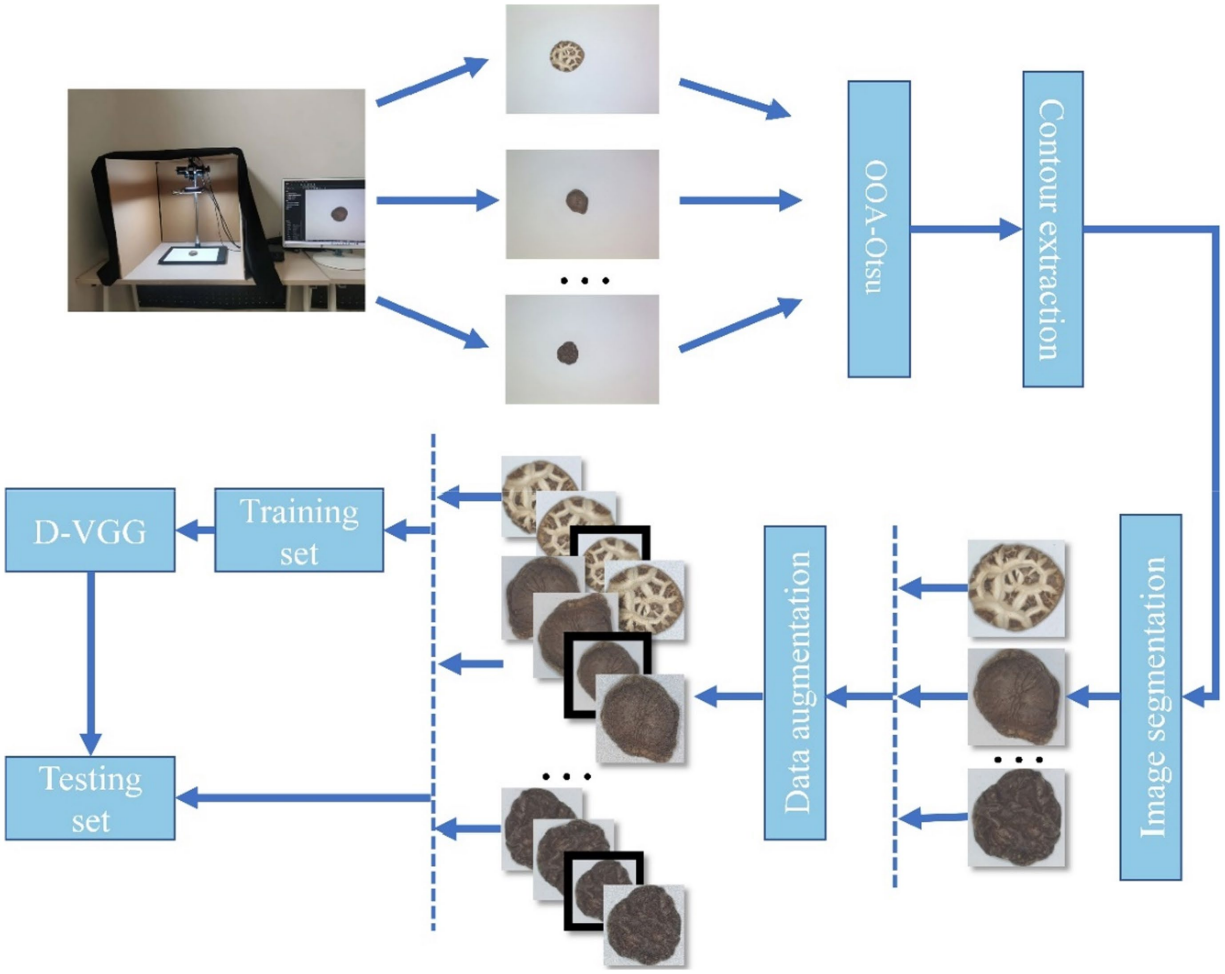
Research flow chart.

In this study, image acquisition and preprocessing are implemented for six types of DSM: Premium Dried Flower Mushrooms (DFM-P), grade 1 Dried Flower Mushrooms (DFM-1), grade 2 Dried Flower Mushrooms (DFM-2), Premium Dried Thick Mushrooms (PDM-TH), grade 1 Dried Thick Mushrooms (DTHM-1), and Premium Dried Thin Mushrooms (PDM-Th). The obtained initial images are enhanced to create the final data set to train a DSM grade recognition model. The overall method flowchart is shown in Figure 1, and the image acquisition and preprocessing steps will be described in detail in the next section.

### 2.1. Image acquisition

**Figure 2:**
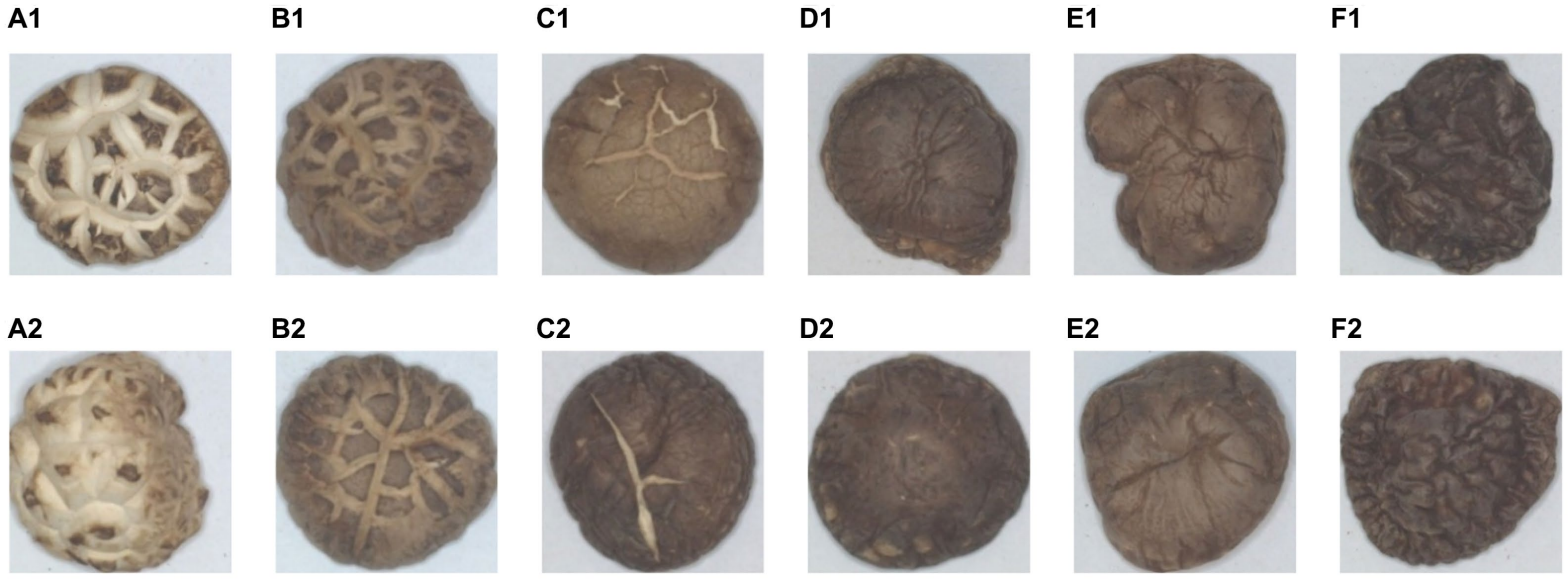
Images of six grades of DSM. **(A1,A2)** DFM-P, **(B1,B2)** DFM-1, **(C1,C2)** DFM-2, **(D1,D2)** PDM-TH, **(E1,E2)** DTHM-1, **(F1,F2)** PDM-Th.

Example images of DSM samples of different grades are shown in Figure 2, where A1,A2 is a DFM-P, B1,B2 is a DFM-1, C1,C2 is a DFM-2, D1,D2 is PDM-TH, E1,E2 is DTHM-1, and F1,F2 is PDM-Th. From Figure 2, it can be seen that different species of DSM have different textural characteristics, and there are some differences between different grades of the same species. It is noteworthy that some DSM species showed relatively slight textural differences between grades, which were difficult to distinguish accurately, for example, PDM-TH and DTHM-1, which brought some challenges and difficulties to the subsequent sorting of DSM grades.

**Figure 3:**
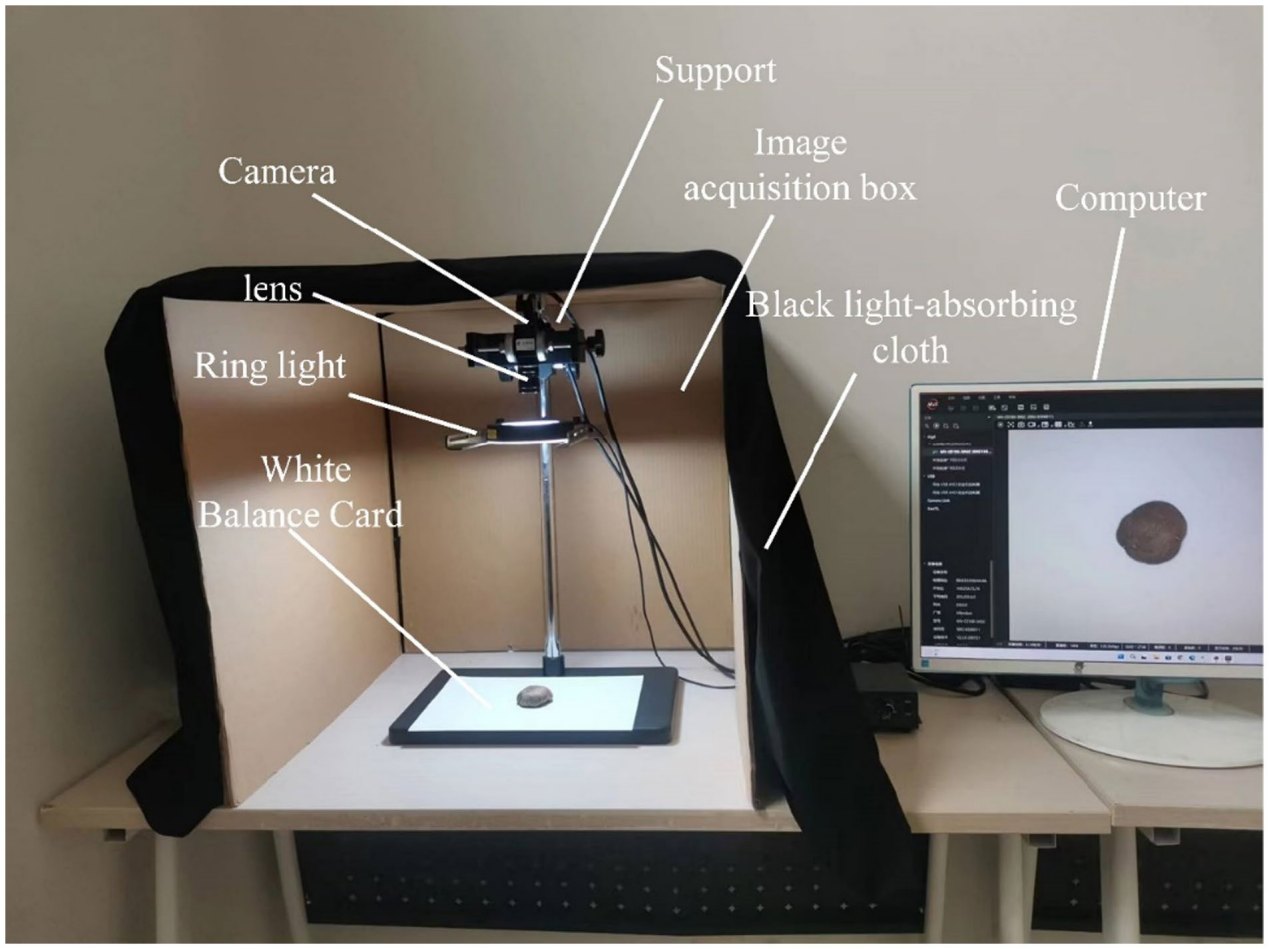
Image acquisition platform.

The image acquisition system is shown in Figure 3. For the DSM grade recognition task, an image acquisition darkroom with a ring light source was designed. The experimental acquisition environment was under the darkroom, which had a size of 60 cm × 60 cm × 60 cm. A professional camera with a black light-absorbing cloth was used to prevent the interference of external light sources, and the light source and camera were fixed on a bracket. To illuminate the targets, a Hikvision Technology industrial high uniform ring light source was used, while the camera model was a Hikvision MV-CE100-30GC 10-megapixel industrial camera, equipped with a MVL-HF1224M-10MP Hikvision optical industrial lens having a focal length of 12 mm. To highlight the color and texture details of the DSM, a white balance card was used as background, and the shooting distance was set at 45 cm. Using the official MVS software provided by Hikvision Robotics Machine Vision, the exposure time was adjusted to 1.818/100 s, the original colors of the image were restored using the gamma correction application that comes with the software, and the automatic white balance was turned on. The open mode was adopted in front of the image acquisition equipment, to allow easy placement and removal of the DSM raw materials. The acquired images were transferred to the computer through a network connection.

A total of 1,500 DSM images were collected in the experiment, and images suffering from acquisition problems such as blurring and incomplete shooting backgrounds were manually screened out. This resulted in 1,355 DSM images after screening, which included 184 DFM-P, 223 DFM-1, 212 DFM-2, 249 PDM-TH, 222 DTHM-1, and 265 PDM-Th images, each with a size of 3,840 × 2,748 pixels. Then, the images were further preprocessed.

### 2.2. Image preprocessing

Continuous identification of various levels of DSM spread on the field assembly line is required; therefore, the shooting field must be much larger than a single mushroom. Then, it does not need to change all the parameters of the image acquisition platform, and the current recognition model and dataset can be directly applied to the subsequent field part detection. The original DSM image size was 3,840 × 2,748 pixels, which was relatively large, while the area occupied by the DSM targets was small, so a large part of the image consisted of irrelevant background pixels. To improve the training and convergence speed of the model, further preprocessing of the originally captured images was necessary to precisely locate the area occupied by the DSM, so that the model could identify the texture features of the DSM and grade them efficiently and correctly. The image preprocessing step was as follows: (1) conversion of images to grayscale; (2) application of improved Otsu’s threshold binarization (OOA-Otsu); (3) DSM outline extraction; (4) cropping of the ROI.

#### 2.2.1. Improved Otsu’s threshold binarization of DSM images

**Figure 4:**
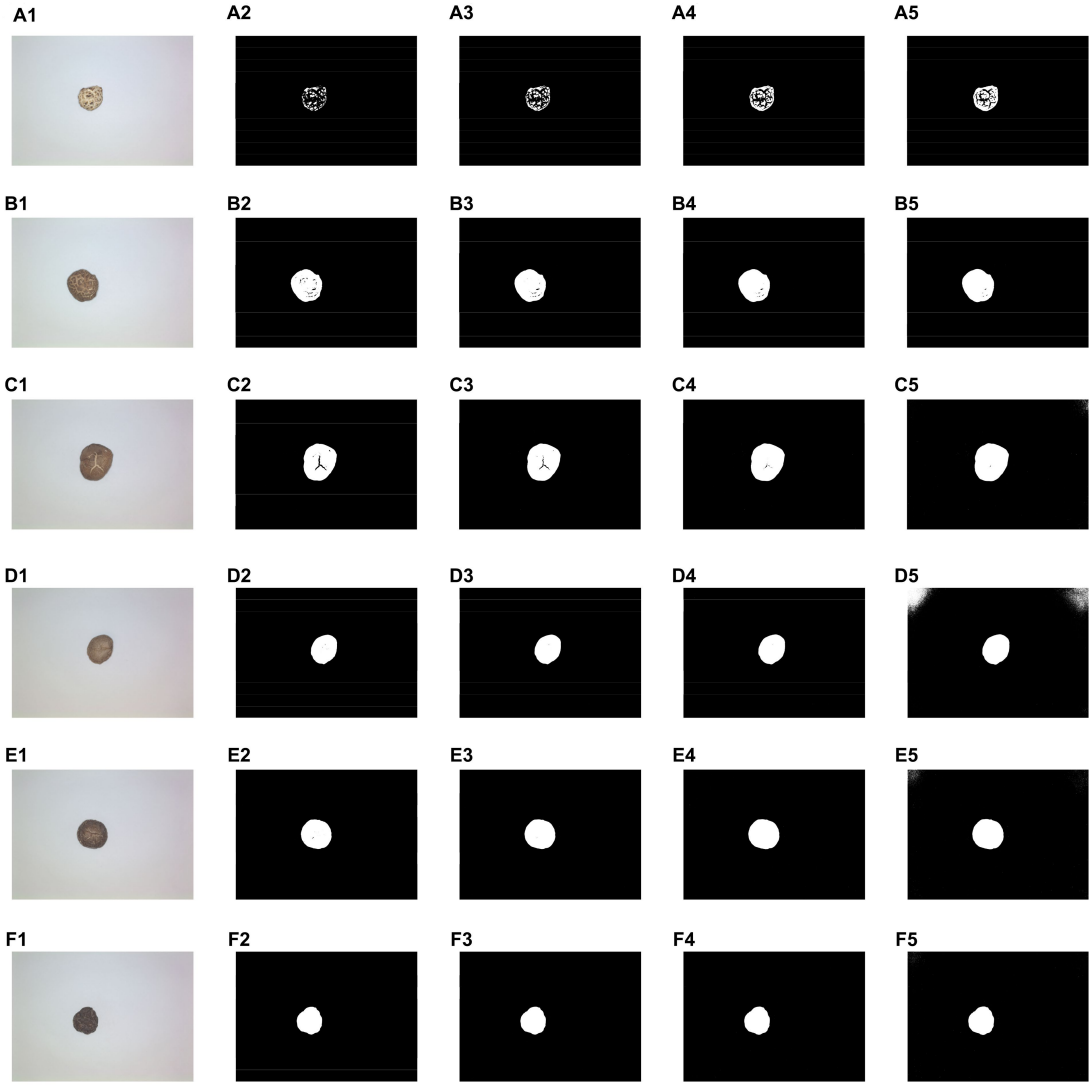
Results of simple threshold binarization under different thresholds. **(A1–F1)** Original image. **(A2–F2)** Threshold 155. **(A3–F3)** Threshold 165. **(A4–F4)** Threshold 175. **(A5–F5)** Threshold 185.

The images were first processed using simple thresholding, which results in the binarization of the images using a direct threshold. By analyzing the grayscale distribution of the different DSM images, four thresholds of 155, 165, 175, and 185 were selected to process the images. Figure 4 shows the results of processing the six DSM grade images using four different thresholds. Among them, A1–F1 represent original DSM images of different grades: A1 corresponds to DFM-P, B1 corresponds to DFM-1, C1 corresponds DFM-2, D1 corresponds to PDM-TH, E1 corresponds DTHM-1, and F1 corresponds to PDM-Th. A2–A5 are the results of the DFM-P image processed using the four thresholds of 155, 165, 175, and 185, respectively, and so similarly for B2–B5, C2–C5, D2–D5, E2–E5, F2–F5. It can be seen that binarizing the images of different DSM grades using different thresholds results in different effects. For the DFM-P image of Figure 4A1, when the global thresholds are 155 (Figure 4A2) and 165 (Figure 4A3), the maximum outer contour appears to be broken, while the contours of other grades of DSM can be accurately identified. For the PDM-TH image of Figure 4D1, when the global threshold is 185 (Figure 4D5), binarization using the threshold results in oversized edges, but the same threshold produces good results in the case of the DFM-P contour (Figure 4A5). Meanwhile, the other DSM grades presented different results under the four thresholds. Therefore, it is difficult to determine a fixed threshold for batch processing of different DSM grades images.

The results obtained for the dataset images of different DSM grades are shown in Table 1. It can be seen that the processing time for different threshold values ranged very little, from 33.21 to 34.15 s. When the threshold value was 175, which yielded the best performance, out of 1,355 samples, 1,299 binarized DSM body contours were complete, and the percentage of complete samples to the total samples was 95.88%; the time required to complete the process was 33.71 s.

**Table 1:** Simple threshold binarization performance evaluation table.

Binarization method	Threshold	Complete samples/total samples	The proportion of complete samples	Execution time
Simple threshold	155	1,251/1,355	92.37%	33.21 s
	165	1,277/1,355	94.26%	34.15 s
	175	1,299/1,355	95.88%	33.71 s
	185	1,290/1,355	95.26%	34.07 s

**Figure 5:**
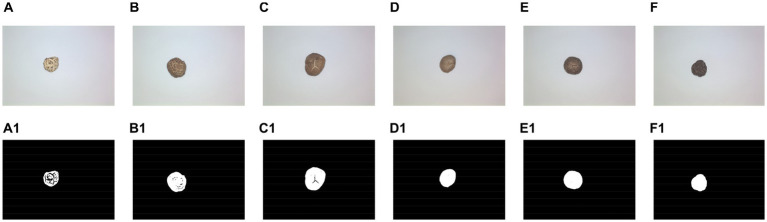
Results of Otsu’s threshold binarization. **(A–F)** Original image. **(A1–F1)** Results of Otsu’s threshold binarization.

Then, Otsu’s threshold binarization method (23, 33) was tested. Figure 5 shows the binarization results of different classes of DSM using Otsu’s method, where A–F are images of the six DSM grades, and A1–F1 are the results with Otsu’s threshold binarization. It can be seen that the maximum contour can be effectively segmented using Otsu’s threshold binarization method. The corresponding performance metrics are shown in Table 2, where the percentage of complete samples to all the samples was 98.37%, and the time required to complete the calculation was 255.28 s.

**Table 2:** Otsu’s threshold binarization performance evaluation table.

Binarization method	Complete samples/total samples	The proportion of complete samples	Execution time
Otsu’s threshold	1,332/1,355	98.37%	255.28 s

**Figure 6:**
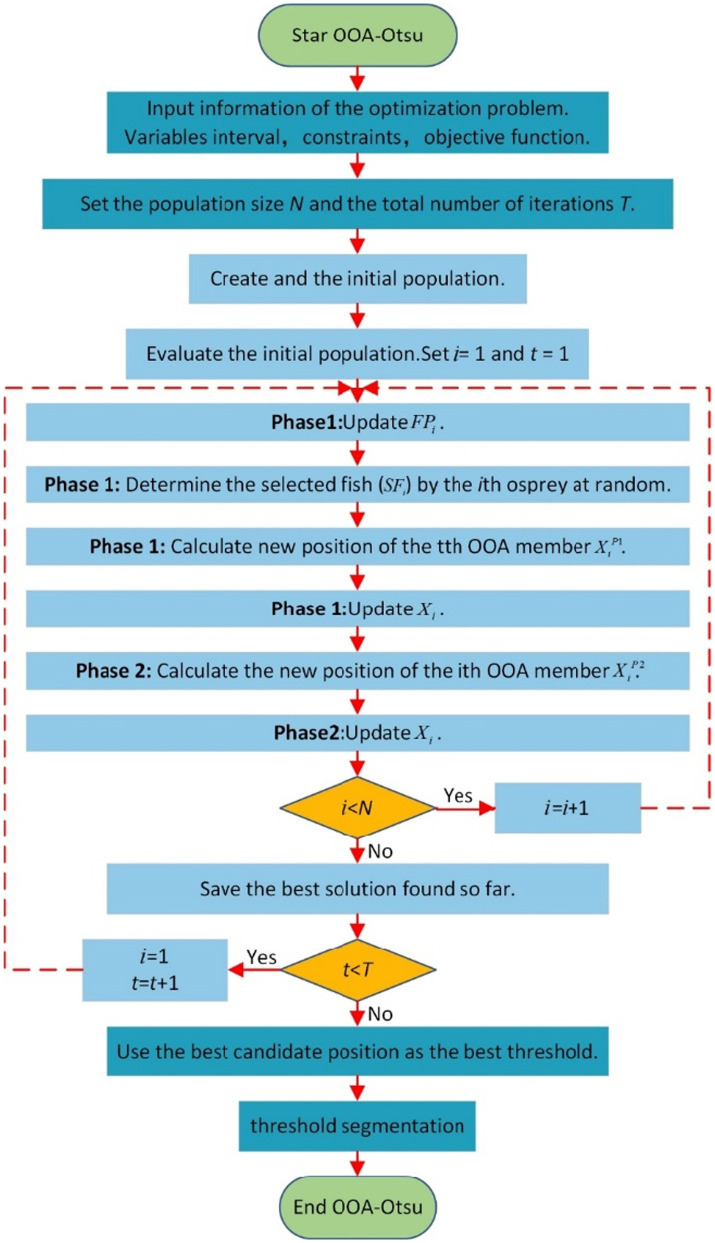
OOA-Otsu algorithm flow chart.

Otsu’s method showed good results in dealing with image binarization for different DSM grades, but the calculation time of the method is long too for practical field applications. The main reason is that the method needs to traverse all the gray levels in the range of 0–255 to calculate the variance. Assuming that the time to calculate the variance for each level is *T*, the total calculation time is 256 T, which greatly reduces the processing efficiency of the DSM image binarization. To ensure that the best threshold value of the image can be obtained while improving efficiency, the osprey optimization algorithm (OOA) (24) was applied to Otsu’s threshold binarization method. The OOA uses the strategy of an osprey hunting fish from the ocean to find the best threshold value. The new method for threshold-based binarization of DSM images is named as OOA-Otsu algorithm. The processing flow of OOA-Otsu algorithm is shown in Figure 6.

In Figure 6, N represents the population size, T represents the maximum number of iterations, i represents the i-th osprey, t represents the current number of iterations, $FP_{i}$ represents the set of fish of the i-th osprey, $SF_{i}$ represents the fish selected by the i-th osprey, $X_{i}$ represents the current position of the i-th fish eagle, $X_{i}^{P1}$ represents the position of the i-th osprey in Phase 1, and $X_{i}^{P2}$ represents the position of the i-th osprey in Phase 2. In this algorithm, the best threshold value in the Otsu’s calculation process is considered as the coordinates $X_{i}$ of the osprey population in the OOA algorithm. The OOA algorithm is used to simulate the predatory behavior of the osprey, where the fitness of each osprey’s position is calculated and inverted. In each iteration, the fitness of the positions is compared, the coordinates $X_{i}$ are updated, and finally the best threshold value is determined, with the aim being to reach the optimal value achieved through Otsu’s exhaustive method.

**Figure 7:**
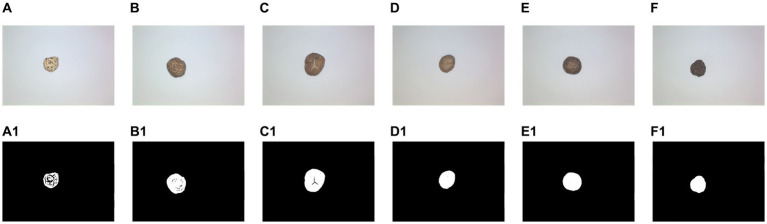
Results of OOA-Otsu for different DSM grades. **(A–F)** Original image. **(A1–F1)** Results of OOA-Otsu.

Figure 7 shows the results of OOA-Otsu algorithm for different DSM grades, where A–F are images of the six DSM grades, and A1–F1 are the processing results with OOA-Otsu. It can be seen that OOA-Otsu can segment the maximum contour of the DSM effectively. The performance analysis of OOA-Otsu is shown in Table 3, where it is shown that the proportion of complete samples to total samples was 99.31% and the time required to achieve this percentage was 52.89 s.

**Table 3:** OOA-Otsu performance evaluation table.

Binarization method	Complete samples/total samples	The proportion of complete samples	Execution time
OOA-Otsu	1,345/1,355	99.31%	52.89 s

The overall performance of the three threshold binarization methods is compared in Table 4. OOA-Otsu proposed in this paper takes the least time with the highest accuracy, and retains more complete contour information when binarizing different DSM images of different grades, thus achieving the best performance.

**Table 4:** Performance evaluation table of different threshold methods.

Binarization method	Complete samples/total samples	The proportion of complete samples	Execution time
Simple threshold	1,299/1,355	95.88%	33.71 s
Otsu’s threshold	1,332/1,355	98.37%	255.28 s
OOA-Otsu	1,345/1,355	99.31%	52.89 s

#### 2.2.2. Dried shiitake mushroom image segmentation

After OOA-Otsu process, all contours of the DSM in the image are obtained. Due to the texture features of some DSM caps, some irrelevant contours may be contained inside the maximum contour of the main body. In addition, there may be some residues around the DSM, and these irrelevant contours are also shown after the threshold segmentation process, so we continue to perform contour filtering to obtain an accurate image of the DSM. In addition, since the classification network requires the same input image length and width, the ROI region is adjusted so that the cropped image has the same length and width to prevent the scaling operation from distorting the DSM image ratio.

**Figure 8:**
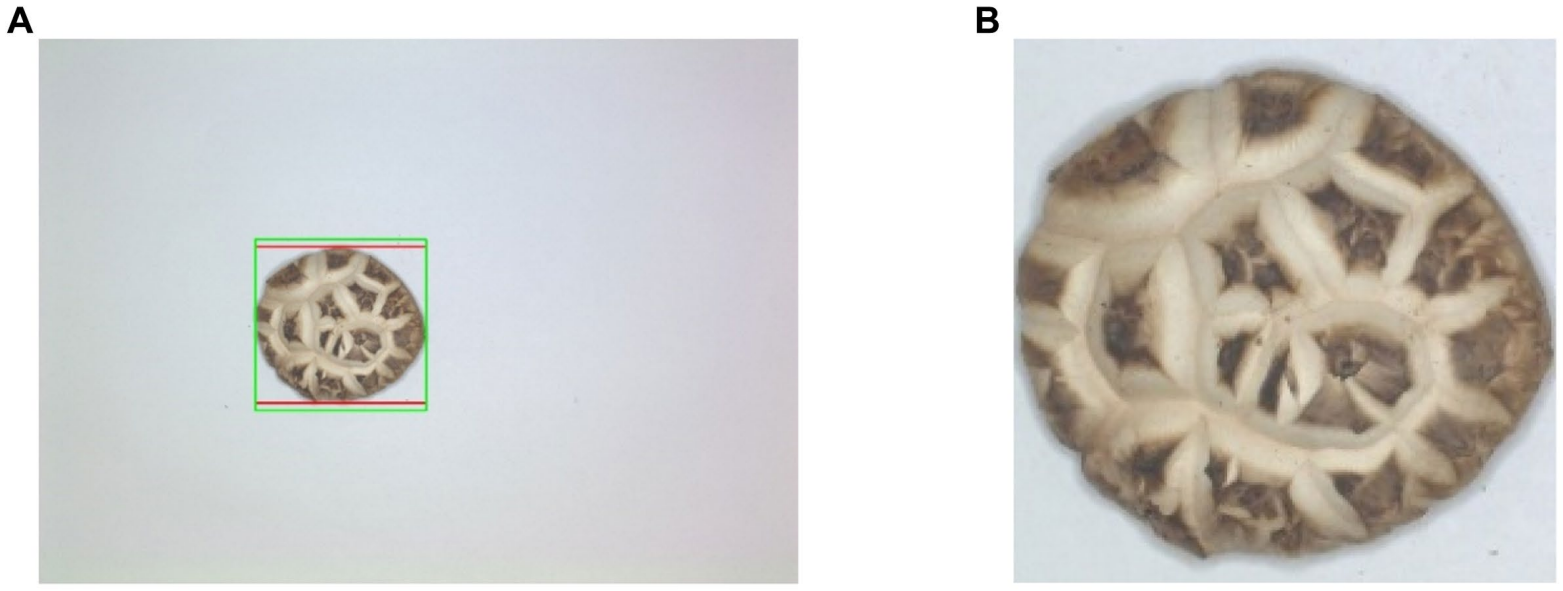
DSM segmentation result. **(A)** Cropped ROI outline. **(B)** Segmented DSM image through ROI.

Figure 8 shows DSM segmentation result. In Figure 8A, the red box indicates the minimum outer rectangle of DSM, while the green box indicates the adjusted minimum outer square. The result of the cropped and resized minimum square ROI is shown in Figure 8B.

### 2.3. Data augmentation

**Figure 9:**
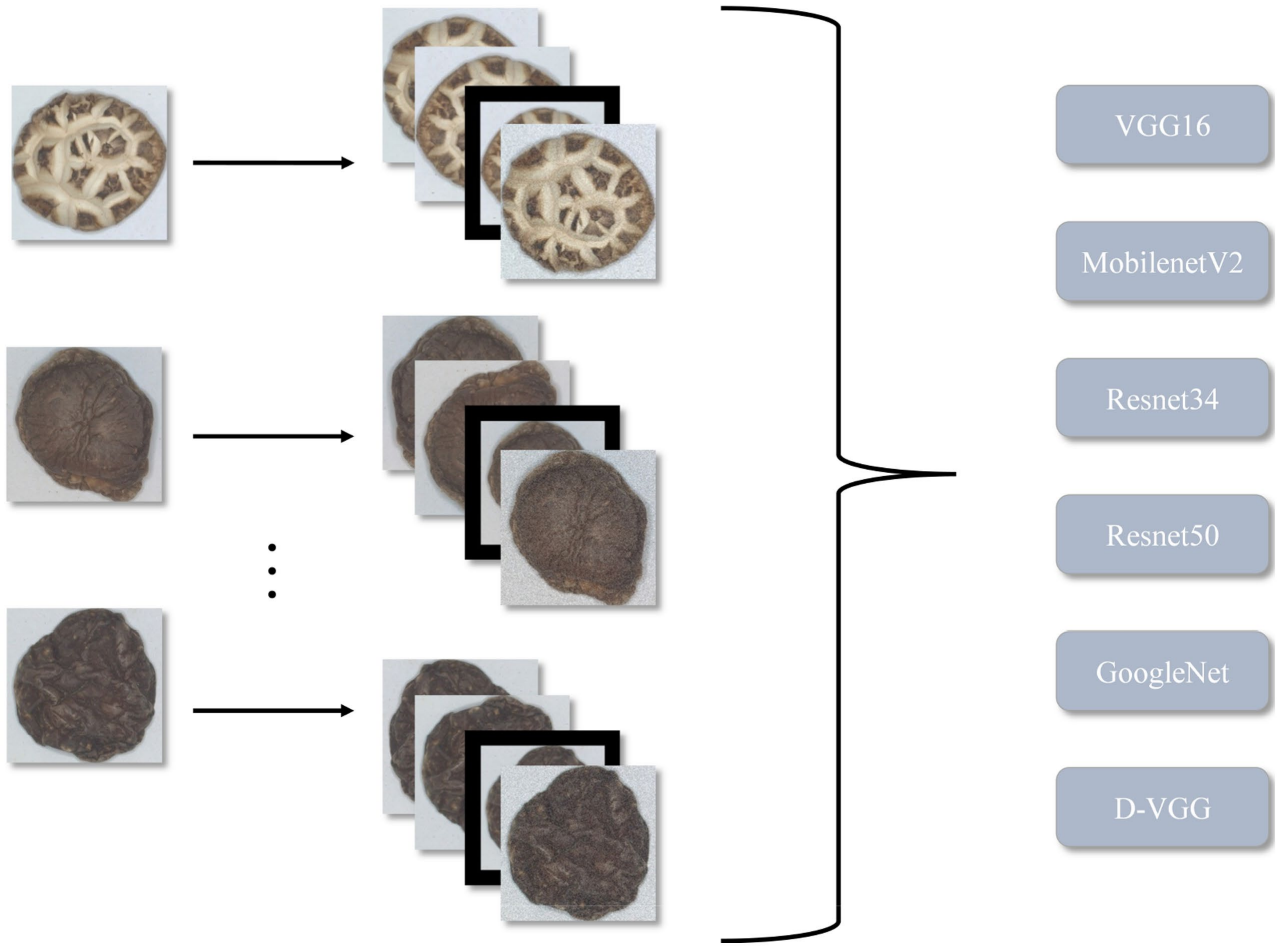
DSM processing effect with image augmentation method.

Cropped DSM images have different pixel sizes. To make the pixel size consistent, we used a double cubic interpolation method to ensure the quality of the scaled image. The pixel size of DSM image was uniformly resized to 224 × 224. To avoid overfitting and improve the generalization ability and recognition accuracy of the model, data augmentation methods were used to increase the number of sample images. Considering the texture characteristics of DSM, the Python library *imgaug* was used to perform horizontal and vertical flips, Gaussian noise addition and equal-scale scaling (25) for the augmentation operations on the existing dataset to generate more samples for model training. Figure 9 shows the DSM processing effect with image augmentation method.

The total number of samples after image augmentation was 6,775, specifically, 920 DFM-P, 1,115 DFM-1, 1,060 DFM-2, 1,245 PDM-TH, 1,110 DTHM-1 and 1,325 PDM-Th. 70% of the final dataset images were used as the training set and the remaining 30% as the test set. The final detailed number of images of different DSM grades is shown in Table 5.

**Table 5:** Details of DSM dataset.

Name	Training set	Testing set
DFM-P	644	276
DFM-1	781	334
DFM-2	742	318
PDM-TH	872	373
DTHM-1	777	333
PDM-Th	928	397
Total	4,744	2,031

## 3. Model construction

### 3.1. Overall D-VGG model framework

**Figure 10:**
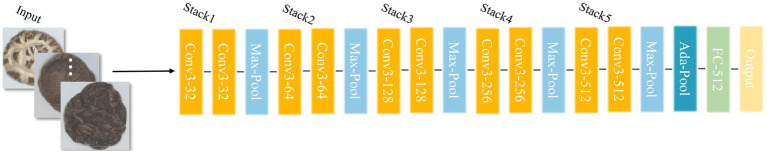
VGG-REV network structure.

**Figure 11:**
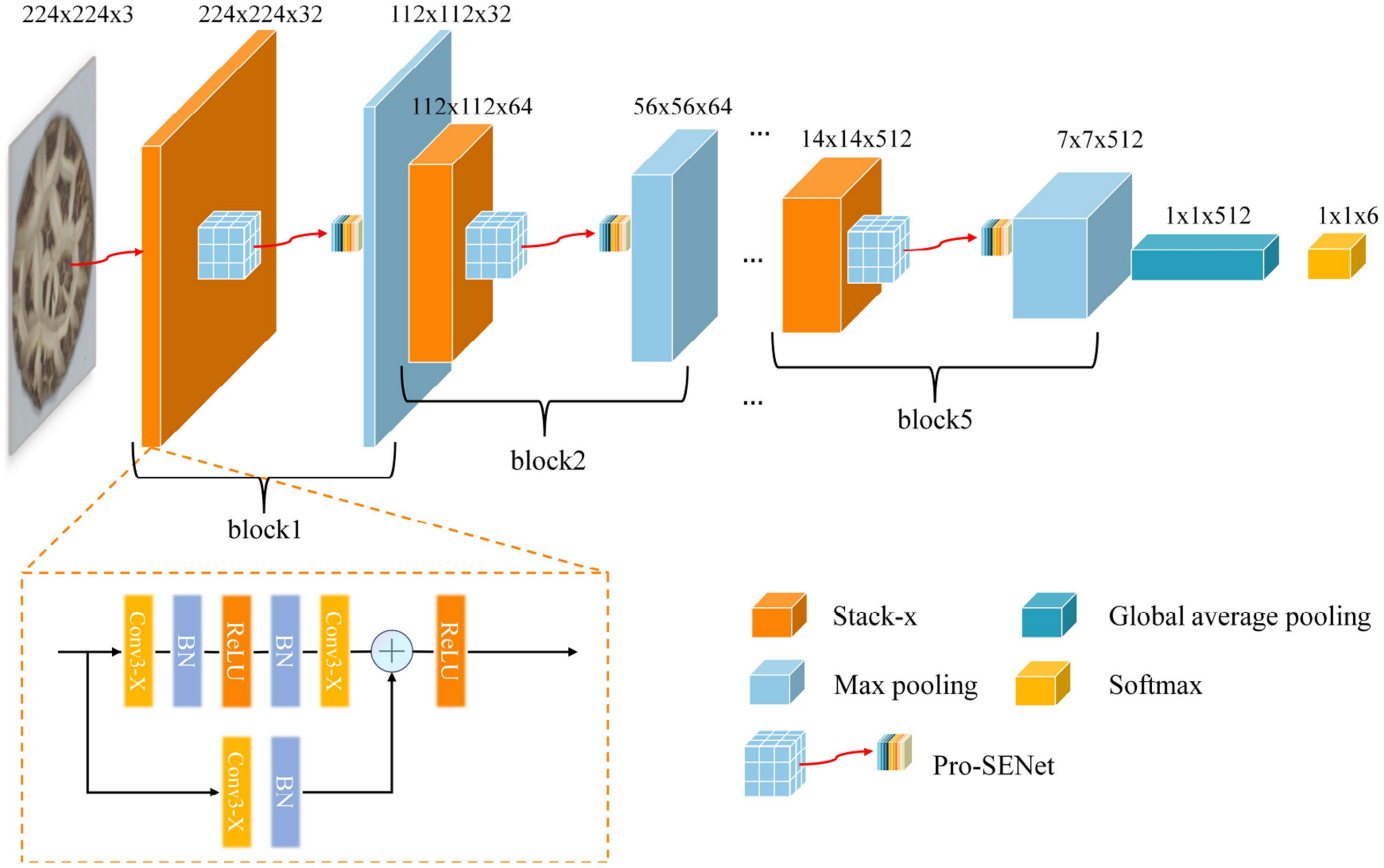
D-VGG network structure.

The VGG16 network model, proposed by the Visual Geometry Group (Oxford University), is one of the classical CNNs and is commonly used for tasks such as image classification, image segmentation, and so on. In this paper, the proposed DSM grade recognition network is based on VGG16. First, the convolutional layer of the VGG16 network was optimized for the DSM grade recognition task through the adoption of a global average pooling layer instead of the fully connected layer. The improved network structure (Figure 10) consisted of five stack modules, a global average pooling layer and a softmax layer, and each stack module contained two sets of convolutions. Second, to enhance the model’s learning of subtle DSM features while accelerating the convergence speed, a residual module and a batch normalization method were introduced in each stacked module of the improved network. To further improve the grading accuracy, an improved channel attention module was proposed, which was introduced into the improved network structure to enhance the weight share of each channel of the DSM image. The final network model was named D-VGG, and its overall structure is shown in Figure 11. It can be seen that the network structure of the D-VGG network consisted of five block layers, a global average pooling layer, and a softmax layer. Among them, each block layer consisted of a stack module, a modified channel attention network, and a pooling layer. Each part of D-VGG will be described in detail next.

### 3.2. Modification of convolutional layer structure and fully connected layer

**Figure 12:**
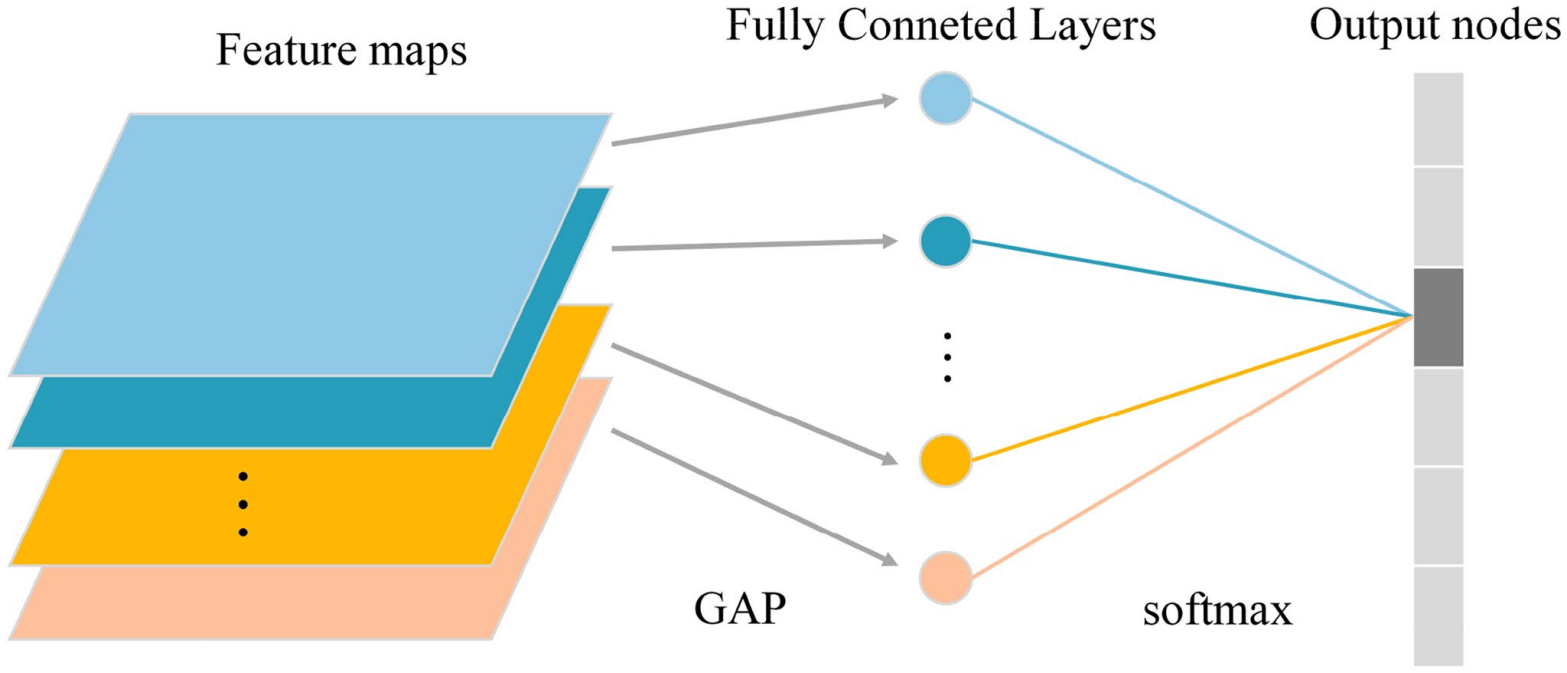
Global average pooling operation flow chart.

Due to the different morphological characteristics and texture features of different grades of DSMs, a convolutional group module with a depth of 32 was added at the very front of the network, which included two sets of convolutional operations. At the same time, the last set of convolutional group modules in the network, with a depth of 512, was removed. The strategy of reducing the depth of convolutional layers aimed to reduce the model complexity and the number of its parameters, reduce the risk of overfitting, and improve the generalization ability of the model in DSM grade recognition tasks. Second, the convolutional groups with depths of 256 and 512 that initially have 3 sets of convolutional layers were adjusted to 2 sets of convolutional layers, consistent with the convolutional groups with depths of 32, 64, and 128. This operation optimized the network structure and facilitates subsequent model building and improvement. Finally, the whole network model had five convolutional groups, each containing two convolutional layers. To better describe their functions and organization, the convolutional groups were renamed as “Stack.” The fully connected layers are the main reason for the large number of parameters of the deep learning network model and the large amount of memory required for training. For the DSM grade recognition task, the final classification was performed across six categories, and it was found that the neurons were too redundant compared to the 1,000 class classification task trained on the ImageNet dataset, which could easily lead to model overfitting. Therefore, a global average pooling operation was performed on the last convolutional pooled feature map to obtain a 512 × 1 × 1 output. Subsequently, the spreading-processed feature maps were input to the softmax layer to complete the final grading result. The flow of the global averaging pooling operation is shown in Figure 12.

The improved VGG neural network model was named VGG-REV. The VGG-REV network layer structure contained five stack modules, a global average pooling layer, and a softmax layer; its VGG-REV neural network structure is shown in Figure 10.

### 3.3. Residual and batch normalization

**Figure 13:**
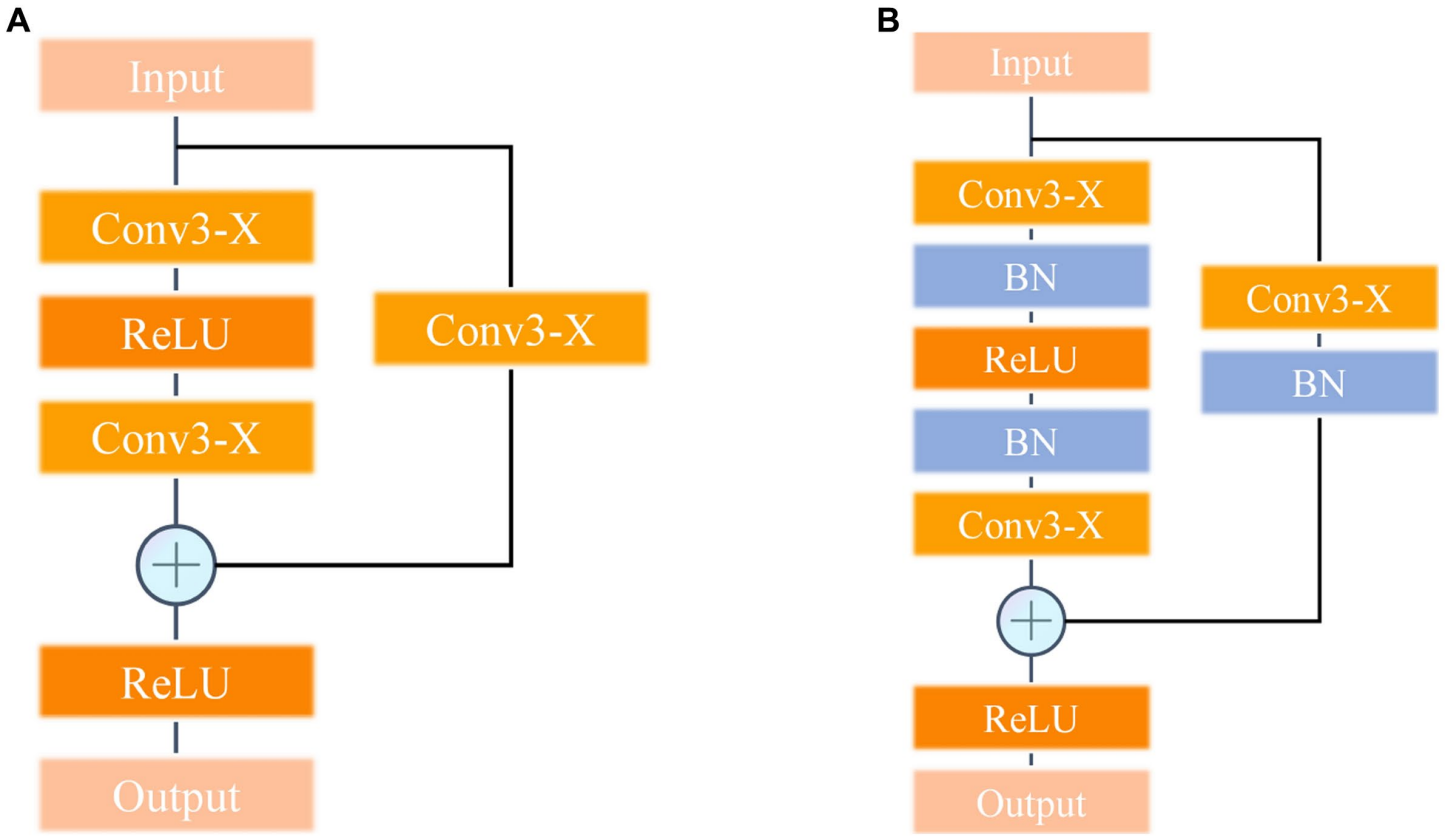
Stack module structure. **(A)** Introduction of residual structure stack module. **(B)** Introducing the stack module with residual structure and batch normalization methods.

The color and texture information of DSMs plays a crucial role in model learning. To extract the subtle features of different DSM grades, the residual structure introduced in the ResNet network is leveraged and a 1 × 1 shortcut branch is added to the input layer of each stacked module of the VGG-REV model to enhance the connection between the texture information of DSM features and the improved neural network. The network structure of a single stack module after the addition of the residual structure is shown in Figure 13A. The main task of the convolutional layer of the CNN is to extract image features. When features from different DSM grades enter the convolutional layer for processing, the distribution of the output data may become uneven due to the update of the convolution parameters, which has an impact on the neural network model. To solve this problem, a batch normalization layer is introduced between each convolutional layer and the activation function to accelerate the convergence of the model and stabilize the training process. During model training, batch normalization uses the mean and standard deviation on small batches of data and maps them to a region with a variance of 1 and a mean of 0 for adjustment, making the output data more stable. The batch normalization calculation process is shown in Eqs. (1) to (5).

First, the mean and variance of the training batch data of each round are calculated:


(1) $$\mu =\frac{1}{m}\overset{m}{\underset{i=1}{\sum }}x_{i}$$

(2) $$\sigma =\frac{1}{m}\overset{m}{\underset{i=1}{\sum }}{\left(x_{i}-\mu \right)}^{2}$$

where $x_{i}$ is an input data sample and m is the total number of input data samples. After obtaining the mean and variance, the batch is regularized to a zero-mean normal distribution with a variance of 1:


(3) $${\hat{x}}_{i}=\frac{x_{i}-\mu }{\sqrt{\sigma^{2}+\varepsilon }}$$

where ε is a tiny positive number used to avoid the case where the denominator is zero and the calculation is meaningless. To avoid the impact of data normalization on the feature distribution, the function is further reconstructed to recover the original feature distribution. The calculation equations are:


(4) $$y_{i}=\gamma_{i}{\hat{x}}_{i}+\beta_{i}$$

(5) $$\gamma_{i}=\sqrt{\mathrm{Var}\left({\hat{x}}_{i}\right)},\beta_{i}=E\left({\hat{x}}_{i}\right)$$

$\gamma_{i}$, $\beta_{i}$ are obtained through the CNN training process, $\mathrm{Var}\left({\hat{x}}_{i}\right)$ is the variance function, and $E\left({\hat{x}}_{i}\right)$ is the mean value function. The batch normalization stem is introduced into the stacked module network structure; the improved structure is shown in Figure 13B, and referred to as the VGG-REV-RES-BN neural network.

### 3.4. Pro-SENet for improved channel attention

In actual production environments, the DSM images have a single background, while the recognition process is inevitably characterized by uneven illumination and the appearance of fine hair point particles, which can introduce a large amount of noise. This noise is passed on to the data used for model training and affects the learning of the feature layer parameters as it propagates within the network, and eventually has an impact on the DSM grade recognition process. To solve this problem, the improved channel attention network Pro-SENet is proposed, which increases the weights associated with the detailed features of DSMs and suppresses the weights of interfering factors by learning each feature’s value automatically. The channel attention (26) network structure contains three main parts: squeezing, stimulation, and feature rescaling. Through the proposed design, the squeezing and stimulation parts of channel attention are improved as follows.

#### 3.4.1. Squeezing

The squeezing operation is performed on input batches of the same samples, and the global average pooling (GAP) and global maximum pooling (GMP) operations are applied on the feature images of dimension *W* × *H* × *C* in the spatial dimension. The feature map *W* × *H* of each channel where GAP is applied is squeezed to a real number with only one global feature, whose output dimension is 1 × 1 × *C*. For each channel processed using GMP, the maximum value of that channel is extracted as its feature map, also with an output dimension of 1 × 1 × *C*. Finally, these two sets of data are averaged and summed to obtain the output of the squeezing operation. It is worth mentioning that replacing the original GAP operation with GAP and GMP operations can alleviate the uneven distribution of GAP weights and further strengthen the weights of the effective channels by the data obtained from GMP. The specific equations are shown in Eqs. (6) to (8).


(6) $$F_{\mathrm{sq}1}\left(u_{c}\right)=\frac{1}{W\times H}\overset{W}{\underset{i=1}{\sum }}\overset{H}{\underset{j=1}{\sum }}u_{c}\left(i,j\right)$$

(7) $$F_{\mathrm{sq}2}\left(u_{c}\right)=\max\left(u_{c}\right)$$

(8) $$z_{c}=F_{\mathrm{sq}2}+\left(F_{\mathrm{sq}1}-F_{\mathrm{sq}2}\right)/2$$

Here, $F_{\mathrm{sq}1}$ denotes the GAP method, $F_{\mathrm{sq}2}$ denotes the GMP method, $u_{c}$ denotes the feature image of the input batch of data, W and H denote the width and height of the feature image, respectively, and (i,j) are the coordinates on the feature image. After the above processing process, the final output channel is $Z_{c}$ and its output is the feature map squeeze result of C.

#### 3.4.2. Stimulation

The result obtained through squeezing linearly varied to change the depth and then is input to the ReLU6 activation function. Compared with the initial ReLU activation function, the ReLU6 function avoids uneven channel learning weights and alleviates the effect of the improved squeezing operation on overweight distribution, thus resulting in more suitable channel weights. Next, the weights of each channel depth are obtained through another linear variation of the initial depth again, and finally the probability distribution is adjusted to the range 0 and 1 using the Sigmoid activation function. The equation is shown in Eq. (9).


(9) $$s=F_{\mathrm{ex}}\left(z_{c},L_{i}\right)=\delta \left(L_{2\sigma }\left(L_{1z_{c}}\right)\right)$$

where $F_{\mathrm{ex}}$ denotes the excitation method, $L_{1}$ and $L_{2}$ are fully connected layers used for linear change processing, σ is the ReLU6 activation function, and δ is the Sigmoid activation function. Finally, a vector S of length C is obtained.

#### 3.4.3. Feature rescaling

The final obtained channel weights S are multiplied with the initial input feature image matrix channel by channel to complete the rescaling of the original feature channel dimensions. The equation is shown in Eq. (10).


(10) $$U_{c}=F_{\mathrm{scale}}\left(u_{c},s_{c}\right)=u_{c}\times s_{c}$$

**Figure 14:**
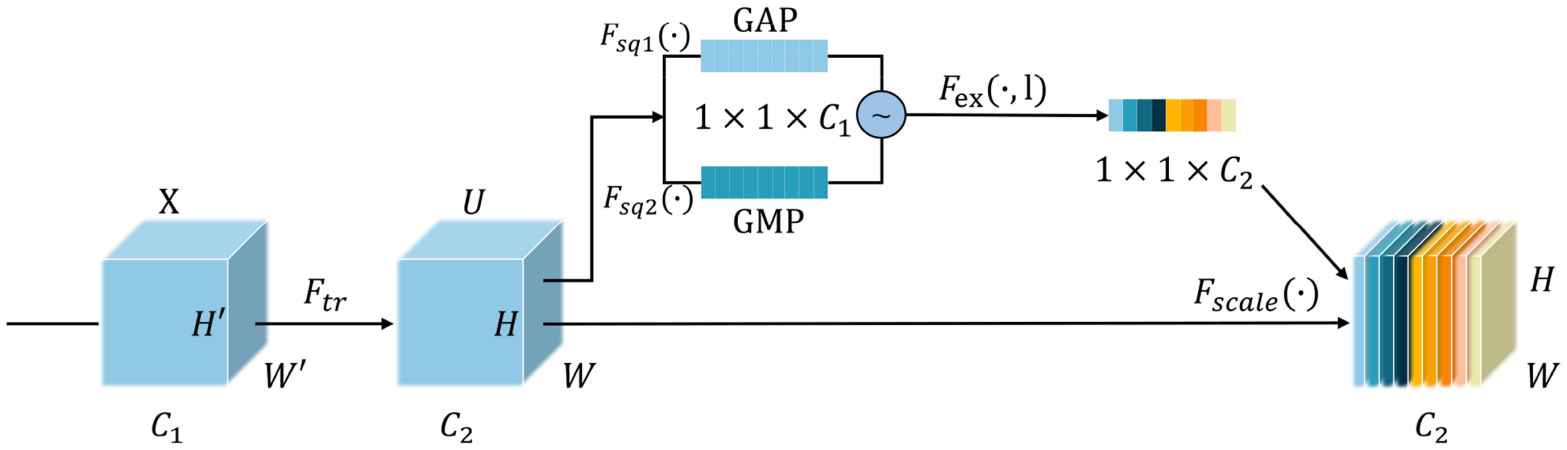
Improved channel attention network structure.

$F_{\mathrm{scale}}$ denotes the feature rescaling method, $u_{c}$ is the feature image of channel as C, and $S_{c}$ is a weight vector of length C. Finally, the output result $U_{c}$ of the feature rescaling process is obtained. The improved channel attention structure is shown in Figure 14.

The improved channel attention network module (Pro-SENet) proposed in this study is a plug-and-play module. Based on the VGG-REV-RES-BN feature extraction network, the improved channel attention module is added to formulate the DSM image feature weighting network to further improve the recognition accuracy of the model. The final neural network model obtained is referred to simply as D-VGG.

## 4. Implementation details

### 4.1. Test platform

The experiments of this study were conducted on the Windows 11 operating system; the GPU model was GeForce RTX 2070 with 8G of video memory, the processor was Intel^®^ Core^™^ i5-12400 CPU at 4.4 GHz, and 16G of running memory were available. The model development environment was Pycharm with Python version 3.9. The model was built using the PyTorch deep learning framework, and the algorithm was accelerated via the CUDA and CUDnn libraries.

### 4.2. Training details

Before inputting the DSM images into the model, the data for each channel were normalized with a mean of 0.5 and a standard deviation of 0.5. Doing so allows different features to have the same scale, thus improving the learning efficiency of the model. In addition, the input training set was randomly scrambled to reduce the effect of image order on the model’s training process. Using the Adam optimizer, the adaptive learning strategy Adagrad combined with the momentum descent algorithm can adapt to the sparse gradient and alleviate the problem of gradient oscillation, so that the model performance was more stable and the model was easy to reach convergence. The initial learning rate (LR) was set to 0.0002, the batch size was set to 32, and the number of iterations to 50. Focal loss function was selected as model loss function. After each epoch, the accuracy of the model was tested with the validation set and validation results generated during that epoch were saved.

### 4.3. Network evaluation

In this study, accuracy (ACC), precision (P), specificity (S), recall (R), F1 score (F1), and avg_metrics (Supplementary Table S1) were used as evaluation metrics to assess the effectiveness of the model. In Supplementary Table S1, TP, TN, FP, FN, $k_{i}$ and $N_{i}$ represent the true positive, true negative, false positive, false negative, evaluation metrics, and the total number of samples of grade *N*, respectively. Among the evaluation metrics, F1 is a composite indicator that incorporates the recall and specificity, and a higher value indicates a better model overall (27). The network evaluation equation is shown in Supplementary Table S1.

## 5. Results

### 5.1. Ablation experiment

To investigate the effects of the different improvements on model performance, a set of ablation experiments were conducted. The same configuration environment with consistent hyperparameters was used for the experiments. The experimental results are shown in Table 6. Among them, D-VGG-REV is the model obtained starting with D-VGG without modifying the convolution and fully connected layers of the network, D-VGG-RES-BN is the model obtained without adding the residual structure and batch normalization, and D-VGG-Pro-SENet is the model without fusing the Pro-SENet network. The comparison of these networks is useful for exploring the impact of each improvement method on the model.

**Table 6:** Comparative results of different models of ablation experiments.

Model	Accuracy	Parameter memory requirement	Model parameter quantity
VGG16	88.97%	512.0 MB	$237.05\times 10^{6}$
D-VGG-REV	94.63%	512.7 MB	$237.24\times 10^{6}$
D-VGG-RES-BN	91.14%	18.1 MB	$4.76\times 10^{6}$
D-VGG-Pro-SENet	94.88%	18.8 MB	$4.94\times 10^{6}$
D-VGG	96.21%	18.9 MB	$4.94\times 10^{6}$

**Figure 15:**
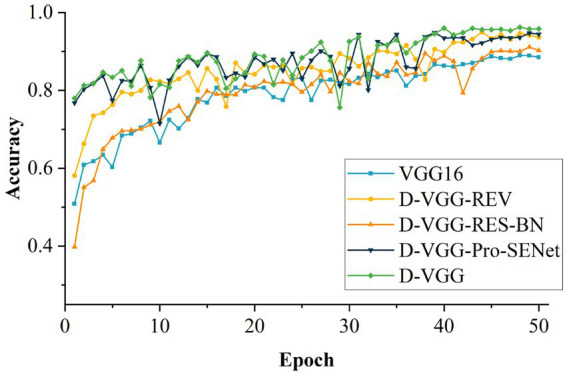
Accuracy of different models for ablation experiments.

From Table 6, it can be seen that the VGG16 model has the worst result in identifying the accuracy of DSM. This is mainly due to the single background of the collected DSM image data, which is too complex for the VGG16 network’s simple model. With the D-VGG-REV model, the accuracy decreases by 1.58 percentage points (pp) compared to D-VGG, and the model memory size increases from 18.1 MB to 512.7 MB. It can be concluded that the modification of the convolutional and fully connected layers of the network results in an improvement of the network’s performance and a significant reduction in the memory required by the model. When the model is D-VGG-RES-BN, the accuracy of the model decreases by 5.07 pp. It can be concluded that the residual module and batch normalization are effective in improving the network performance. This is mainly because the level of details in the feature information of the DSM gradually decreases as model depth increases, and the introduction of the residual module solves this problem. In addition, batch normalization speeds up the training and convergence of the network and prevents the adverse effects of uneven data on the model, thus resulting in an improvement in recognition accuracy. The D-VGG-Pro-SENet model’s accuracy decreases by 1.33 pp. which is mainly because the model with channel attention can learn the feature channels of the DSM more effectively and adaptively, boosting the channel weights that are favorable for model learning. Figure 15 shows the accuracy comparison of each model for 50 iterations. In summary, the improved method proposed in this study achieved a large performance improvement relative to the VGG16 network.

### 5.2. Influence of learning rate and batch size on model training

The learning rate (LR) and the batch size are crucial parameters in deep learning model training. To determine the optimal initial LR and batch size values, experiments were conducted and the effects of different values for these hyperparameters were compared.

#### 5.2.1. Effect of learning rate on model training

The batch size was first set to 32 and experiments were conducted on the effect of different LRs on the model’s performance. The LR was compared in terms of order of magnitude and of different values of the same order. The experimental results are shown in Table 7. As the LR ranged from $10^{-3}$ to $10^{-5}$, the highest accuracy and the smallest loss value were obtained for the values of the $10^{-4}$ order of magnitude. This is mainly because the initial LR was set at the order of $10^{-3}$, which is too large, resulting in the network not being able to converge to the optimal solution stably, jumping larger distances in the parameter space and missing the optimal global minimum. In contrast, when the initial LR was set at an order of magnitude of $10^{-5}$, the gradient of the network decreased too slowly, as the too-small LR resulted in the optimal result not being obtained after iteration limit had been reached. Therefore, it can be concluded that the network model achieved its highest accuracy and best training results for LRs at the $10^{-4}$ order of magnitude.

**Table 7:** Performance comparison of models using different learning rates.

Experiment code	Learning rate	Accuracy	Loss value in training	Avg. specificity	Avg. precision	Avg. recall	Avg. *F*1 Score
1	0.001	93.99%	0.0292	98.80%	93.95%	93.92%	93.93%
2	0.0001	94.63%	0.0087	98.90%	94.85%	94.55%	94.70%
3	0.00001	90.00%	0.0712	99.98%	90.05%	89.62%	89.83%
4	0.002	92.76%	0.0217	98.53%	92.98%	92.55%	92.77%
5	0.0002	96.21%	0.0054	99.25%	96.18%	96.33%	96.26%
6	0.00002	92.37%	0.0367	98.47%	92.22%	92.30%	92.26%
7	0.003	93.89%	0.0177	98.78%	94.02%	93.68%	93.85%
8	0.0003	95.67%	0.0126	99.13%	95.68%	95.70%	95.69%
9	0.00003	92.81%	0.0223	98.57%	92.78%	92.82%	92.80%

**Figure 16:**
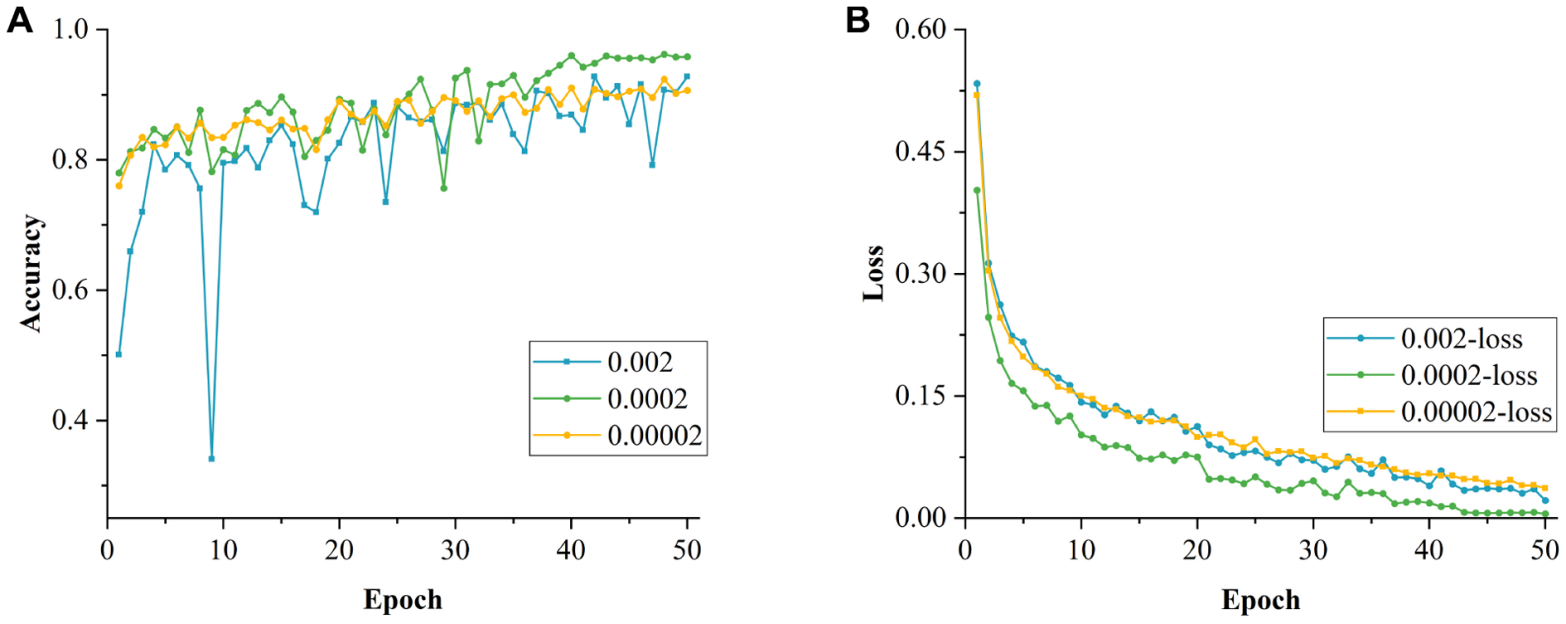
Longitudinal comparison of models with different learning rates. **(A)** Accuracy of models with different learning rates in the longitudinal direction. **(B)** Loss values for models with different learning rates in the longitudinal direction.

**Figure 17:**
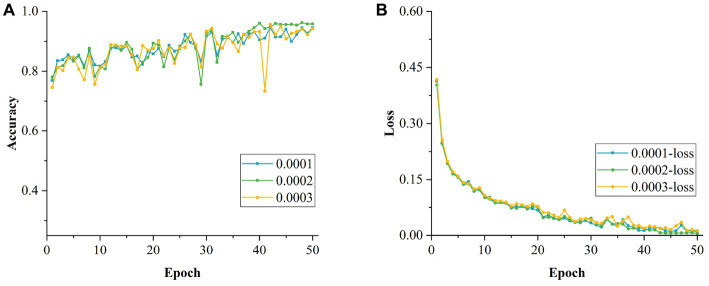
Cross-sectional comparison of models with different learning rates. **(A)** Accuracy of models with different learning rates in the cross-section. **(B)** Loss values for models with different learning rates in the cross-section.

Figures 16A,B show the changes in accuracy and loss values during the training of the model for different initial LRs magnitudes, respectively. Based on this, a cross-sectional comparison was conducted by varying the initial LR between 0.0001 and 0.0003. From Table 7, it can be concluded that the optimal initial LR of this model was 0.0002, resulting in the highest accuracy rate of 96.21% with the smallest training loss value of 0.0054. Figures 17A,B show the changes in accuracy and loss values during the training of the model for different LR values at the $10^{-4}$ order of magnitude.

#### 5.2.2. Effect of different batch sizes on model training

**Figure 18:**
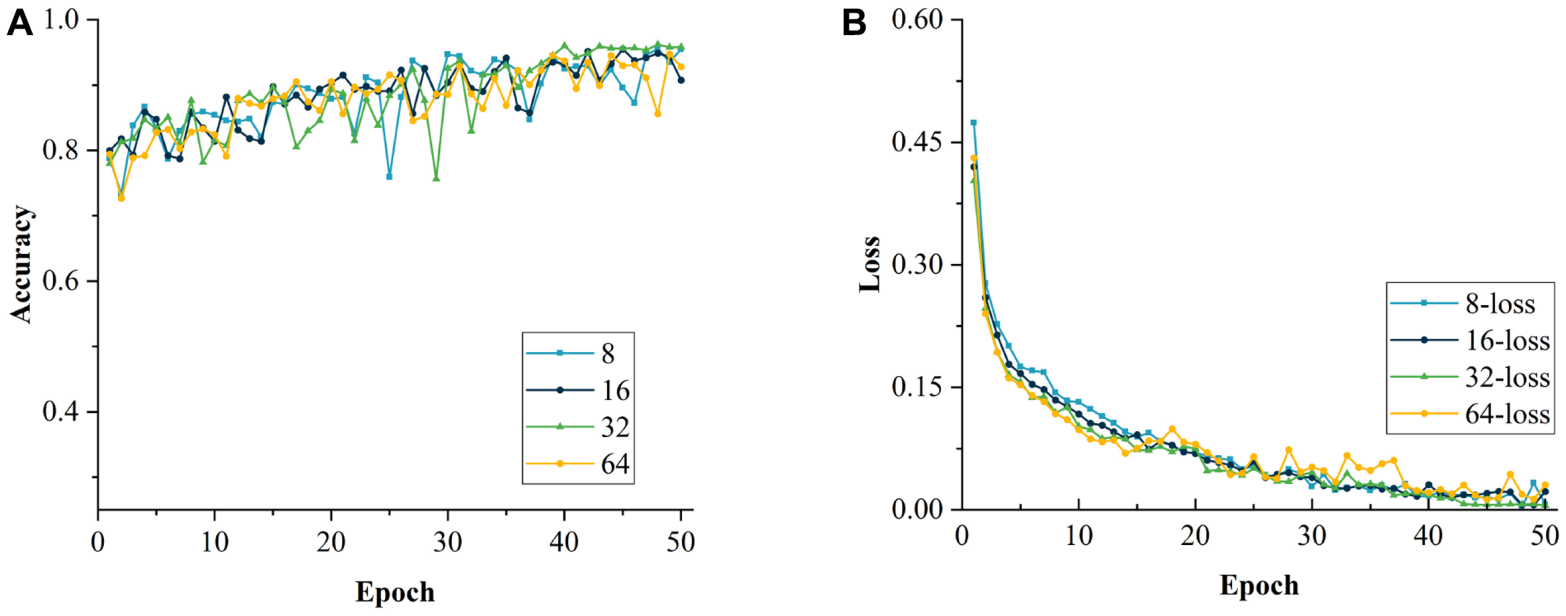
Comparison of models with different batch size. **(A)** Accuracy of models with different batch size. **(B)** Loss values for models with different batch size.

After determining the optimal initial LR, the LR was fixed at 0.0002. The network performance was evaluated for different batch sizes. The size of the batch is crucial for the improvement of the model performance. The values tested were 8, 16, 32 and 64, which were incremented by a factor of $2^{x}$ (*x* = 1, 2, 3, 4) to observe the impact of different batch sizes more intuitively. As can be seen from Table 8, when the batch size was set to 64, the accuracy of the model recognition was the lowest, which is mainly because this large setting reduced the generalization ability of the model and affected its performance. On the contrary, when the batch size was set to 8, the accuracy of the model was also relatively low, mainly because a small value led to a large variation between adjacent batches, which affected the convergence of the model negatively. As the batch size was increased to 16, the recognition accuracy of the model improved, but it still did not reach the optimal effect, and the model was still in a locally optimal state. Through the experiments, it can be concluded that the model has the highest accuracy and the best performance with the batch size set to 32. Figures 18A,B show the changes in accuracy and loss value during the training of the model with different batch sizes, respectively.

**Table 8:** Performance comparison of models using different batch size.

Experiment code	Batch size	Accuracy	Loss value in training	Avg. specificity	Avg. precision	Avg. recall	Avg. *F*1 score
1	8	95.52%	0.0062	99.13%	95.73%	95.68%	95.71%
2	16	95.57%	0.0040	99.02%	95.42%	94.80%	95.11%
3	32	96.21%	0.0054	99.25%	96.18%	96.33%	96.26%
4	64	94.73%	0.0129	98.95%	94.70%	94.58%	94.64%

### 5.3. D-VGG: comparison with other networks

To verify the effectiveness of the network proposed in this paper, the following models were chosen as baseline models: VGG16 (28), GoogleNet (29), ResNet34 (30), ResNet50 (31), and MobileNetV2 (32).

**Figure 19:**
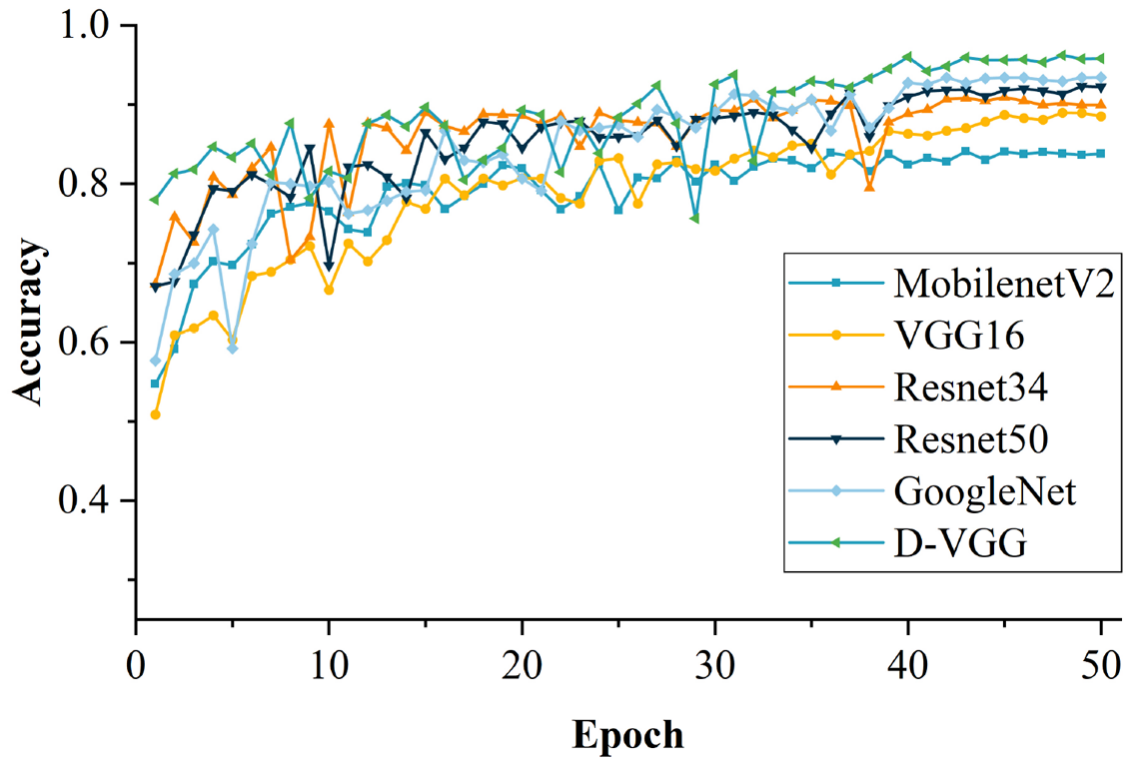
Comparison of the accuracy of different models.

The accuracy variation of each model during the training process after 50 training iterations is shown in Figure 19. The performance of each model was evaluated, and the evaluation results are shown in Table 9. From Table 9, it is evident that the proposed D-VGG model outperforms other similar deep-learning network models in all indices. In terms of accuracy rate, the D-VGG model proposed in this paper achieved 96.21%. The next best model is GoogleNet, with an accuracy of 93.45%. GoogleNet used the Inception module, which incorporated multiple scale features and enabled the model to achieve better performance. The accuracy of ResNet 50 and ResNet 34 was 92.31% and 90.89%, respectively. ResNet employed a residual structure, which successfully solved the degradation problem that occurred in deep networks, and also mitigated the issue of gradient disappearance and explosion that resulted from increasing network depth. However, MobilenetV2 and the baseline VGG16 model performed poorly at 84.78% and 88.97%, respectively. MobilenetV2 had a relatively shallow structure and thus lost some characterization ability when dealing with DSM images with complex features. The VGG16 network can capture finer-grained features of DSM images, but cannot exploit the spatial information and is prone to overfitting. In summary, the D-VGG network proposed in this paper performs the best on the DSM grade recognition task.

**Table 9:** Performance comparison of different models.

Model	Accuracy	Avg. specificity	Avg. precision	Avg. recall	Avg. *F*1 score	Execution time
VGG16	88.97%	97.78%	89.05%	88.87%	88.96%	97.49 ms
MobileNetV2	84.09%	96.82%	84.48%	83.82%	84.15%	24.13 ms
ResNet34	90.89%	98.17%	91.03%	90.60%	90.82%	32.50 ms
ResNet50	92.32%	98.45%	92.32%	92.15%	92.23%	54.65 ms
GoogleNet	93.45%	98.68%	93.53%	93.37%	93.45%	26.10 ms
D-VGG	96.21%	99.25%	96.18%	96.33%	96.26%	46.77 ms

In addition to considering performance metrics such as model accuracy, specificity, recall, and *F*1 score, the complexity of the model in terms of time taken to complete a task is also one of the important considerations. Excessive complexity can affect model deployment and practical field applications, especially in industrial production, where a fast response time may have a large impact in productivity. The average inference time for the total test set of 2031 DSM images was calculated, and the results are shown in Table 9. It can be seen that the D-VGG single image prediction time is only 46.77 ms, which is equivalent to processing 21 images per second, and meets the requirements of the actual field.

## 6. Conclusion

For solving the problem of DSM grade recognition in the real field, this study realized an experimental platform construction, image processing and DSM dataset establishment. The DSM grade recognition network model D-VGG was then proposed and its performance was evaluated. The innovative points of this paper are as follows:

1. An Otsu’s threshold binarization based on the osprey optimization algorithm is designed, which can extract the DSM contours efficiently and accurately. This algorithm uses the Osprey optimization algorithm as an optimization tool, which is applied to Otsu’s algorithm to achieve adaptive threshold binarization of the image. Contour screening and ROI region cropping steps are further performed to segment of the DSM images. The complete DSM image is obtained by extending the ROI.
2. A D-VGG network model for DSM grade recognition is proposed. Using the VGG16 network as the main framework, the convolutional layer of the network is optimized and the GAP is used instead of the fully connected layer of the network, which accelerates the training of the model and decreases the number of network parameters. The residual module and batch normalization are introduced to enhance the network’s ability to recognize the detailed features of different DSM grades. An improved channel attention module is also proposed and introduced into the model for effective learning of channel weights.

The network model proposed realizes a effective classification of different DSM grades. However, there are some limitations in this study. First, the dataset used in this paper is enhanced and the number of original images is relatively small. Second, the size information of different grades of DSM is not utilized during the model training process, and the size features of DSM are lost. Follow-up work should consider the following aspects:

1. The number of samples of different DSM grades should be expanded, so that the neural network can learn and understand the characteristics of different grades more fully, which will result in an improved generalization ability.
2. The effects of geometric features such as length, width, area, and aspect ratio of different types of DSM on the grading results should be studied, and these features should be analyzed by the network together with image information to improve the recognition accuracy.
3. This study was conducted in the laboratory, and the method should be subsequently validated in a practical application scenario.

## Data availability statement

The raw data supporting the conclusions of this article will be made available by the authors, without undue reservation.

## Author contributions

LW and PD researched the background, proposed the methodology, designed the experiment, and wrote and revised the manuscript. PD and KJ participated in the dataset acquisition and designed the model testing experiments. QW and QN set up the experimental sites and participated in the overall scheme proposal and model modification. All authors contributed to the article and approved the submitted version.
