# Supplementary material for: Dried shiitake mushroom grade recognition using D-VGG network and machine vision
Source: Front Nutr. 2023 Oct 18;10:1247075. doi: 10.3389/fnut.2023.1247075 (PMC10618359; doi:10.3389/fnut.2023.1247075)
Supplement: Supplementary file 1 [file Data_Sheet_1.docx]

Supplementary Material

**Supplementary Table 1** | Table of evaluation indicators.

| NO. | Evaluation indicators | Calculating formulas |
| --- | --- | --- |
| 1 | Accuracy rate (ACC) | $ACC=\frac{TP+TN}{TP+TN+FP+FN}$ |
| 2 | Precision rate (P) | $P=\frac{TP}{TP+FP}$ |
| 3 | Specificity rate (S) | $S=\frac{TN}{TN+FP}$ |
| 4 | Recall rate (R) | $R=\frac{TP}{TP+FN}$ |
| 5 | F1 score (F1) | $F1=2\times\frac{P\times R}{P+R}$ |
| 6 | Average metrics ($avg\_metrics$) | $avg\_metrics=\frac{\sum_{i=1}^{i=6} k_{i}}{\sum_{i=1}^{i=6} N_{i}}\times100\%$ |
